# A Peculiar CLL Case with Complex Chromosome 6 Rearrangements and Refinement of All Breakpoints at the Gene Level by Genomic Array: A Case Report

**DOI:** 10.3390/jcm12124110

**Published:** 2023-06-17

**Authors:** Michele Cennamo, Davide Sirocchi, Carolina Giudici, Marzia Giagnacovo, Guido Petracco, Daniela Ferrario, Simona Garganigo, Angela Papa, Emanuela Veniani, Alessandro Squizzato, Lucia Del Vecchio, Carlo Patriarca, Michelarcangelo Partenope, Piergiorgio Modena

**Affiliations:** 1 Department of Translational Medical Sciences, University of Naples “Federico II”, 80131 Naples, Italy; 2 Clinical Pathology and Microbiology Unit, Laboratory Analysis, ASST Lariana, Hospital Sant’Anna, 22100 Como, Italy; simona.garganigo@asst-lariana.it (S.G.); angela.papa@asst-lariana.it (A.P.); emanuela.veniani@asst-lariana.it (E.V.); michelarcangelo.partenope@asst-lariana.it (M.P.); 3 General Medicine Unit, ASST Lariana, Hospital Sant’Anna, 22100 Como, Italy; davide.sirocchi@asst-lariana.it (D.S.); alessandro.squizzato@asst-lariana.it (A.S.); 4 Genetics Unit, ASST Lariana, Hospital Sant’Anna, 22100 Como, Italy; carolina.giudici@asst-lariana.it (C.G.); marzia.giagnacovo@asst-lariana.it (M.G.); 5 Pathological Unit, ASST Lariana, Hospital Sant’Anna, 22100 Como, Italy; guido.petracco@asst-lariana.it (G.P.); daniela.ferrario@asst-lariana.it (D.F.); carlo.patriarca@asst-lariana.it (C.P.); 6 Department of Medicine and Surgery, Research Centre on Thromboembolic Disorders and Antithrombotic Therapies, University of Insubria, 21110 Varese, Italy; 7 Department of Nephrology and Dialysis, ASST Lariana, Hospital Sant’Anna, 22100 Como, Italy; lucia.delvecchio@asst-lariana.it

**Keywords:** chronic lymphocytic leukemia, CLL, autoimmune hemolytic anemia, AIHA, complex karyotype, FISH, chromoanagenesis, rituximab, acalabrutinib

## Abstract

Introduction: Chronic lymphocytic leukemia (CLL), the most common leukemia in Western countries, is a mature B-cell chronic lymphoproliferative disorder characterized by the accumulation of neoplastic CD5+ B lymphocytes, functionally incompetent and usually monoclonal in origin, in bone marrow, lymph nodes and blood. Diagnosis occurs predominantly in elderly patients, with a median age reported between 67 and 72 years. CLL has a heterogeneous clinical course, which can vary from indolent to, less frequently, aggressive forms. Early-stage asymptomatic CLL patients do not require immediate therapeutic intervention, but only observation; treatment is necessary for patients with advanced disease or when “active disease” is observed. The most frequent autoimmune cytopenia (AIC) is autoimmune haemolytic anaemia (AHIA). The main mechanisms underlying the appearance of AIC in CLL are not fully elucidated, the predisposition of patients with CLL to suffering autoimmune complications is variable and autoimmune cytopenia can precede, be concurrent, or follow the diagnosis of CLL. Case presentation: A 74-year-old man was admitted to the emergency room following the finding of severe macrocytic anaemia during blood tests performed that same day, in particular the patient showed a profound asthenia dating back several months. The anamnesis was silent and the patient was not taking any medications. The blood examination showed an extremely high White Blood Cell count and findings of AIHA in CLL-type mature B-cell lymphoproliferative neoplasia. Genetic investigations: Conventional karyotyping was performed and it obtained a trisomy 8 and an unbalanced translocation between the short arm of chromosome 6 and the long arm of chromosome 11, concurrent with interstitial deletions in chromosomes 6q and 11q that could not be defined in detail. Molecular cytogenetics (FISH) analyses revealed Ataxia Telangiectasia Mutated (ATM) monoallelic deletion (with loss of ATM on derivative chromosome 11) and retained signals for TP53, 13q14 and centromere 12 FISH probes. TP53 and IGHV were not mutated. Array-CGH confirmed trisomy of the entire chromosome 8 and allowed us to resolve in detail the nature of the unbalanced translocation, revealing multiple regions of genomic losses on chromosomes 6 and 11. Discussion: The present case report is an unusual CLL case with complex karyotype and refinement of all breakpoints at the gene level by the genomic array. From a genetic point of view, the case under study presented several peculiarities. Conclusions: We report the genetic findings of a CLL patient with abrupt disease onset, so far responding properly to treatments despite the presence of distinct genetic adverse traits including ATM deletion, complex karyotype and chromosome 6q chromoanagenesis event. Our report confirms that interphase FISH alone is not able to provide an overview of the whole genomic landscape in selected CLL cases and that additional techniques are required to reach an appropriate cytogenetic stratification of patients.

## 1. Introduction

Chronic lymphocytic leukemia (CLL), the most common leukemia in Western countries, is a mature B-cell chronic lymphoproliferative disorder characterized by the accumulation of neoplastic CD5+ B lymphocytes, functionally incompetent and usually monoclonal in origin, in bone marrow, lymph nodes and blood [1,2,3].

Diagnosis occurs predominantly in elderly patients, with a median age reported between 67 and 72 years, where men are more frequently affected than women (ratio of 1.7:1) [4].

CLL has a heterogeneous clinical course, which can vary from indolent to, less frequently, aggressive forms.

In contrast to other B-cell malignancies, immune dysregulation is a constant feature of CLL that is associated with a high prevalence of infections, autoimmune phenomena, and secondary malignancies [5,6,7,8,9].

The most frequent autoimmune cytopenias (AICs) are autoimmune hemolytic anemia (AIHA, 7–10%) and immune thrombocytopenia (ITP, 1–5%), while pure red cell aplasia (<1%) and autoimmune neutropenia (0.17%) are unusual. AIHA can appear in up to a third of patients with CLL during the course of their disease, while in 10–15% of cases is already present at diagnosis [10,11,12,13,14,15,16,17].

The main mechanisms underlying the appearance of AIC in CLL are not fully elucidated and the predisposition of patients with CLL to suffering autoimmune complications is variable. For example, some studies have shown a higher incidence of AIC in patients with an advanced clinical stage, especially in those with spleen infiltration, suggesting that the spleen may act as the site of the presentation of antigens to the T cells. There are, however, some other studies in which no association was found between AIC and survival [9,10,11,12,13,18,19,20,21,22]. The reasons are unclear, but they could be explained with patients’ characteristics, better treatment strategies for CLL and expert management of AIC.

AIHA is defined according to autoantibody thermal characteristics [23,24,25]. From a clinical point of view, AIHA is a heterogeneous condition caused by autoantibodies directed against the red blood cells (RBCs), with or without complement activation. The mechanisms for RBC destruction may be represented by intravascular or extravascular hemolysis.

Autoimmune cytopenia can precede, be concurrent, or follow the diagnosis of CLL. Non-active CLL and AIC should be treated according to current AIHA and ITP guidelines. However, active CLL-related AIC presents some challenges regarding its diagnosis, prognosis, and management. The diagnosis requires a high degree of suspicion as CLL-related AIC cannot manifest in their typical clinical and laboratory features; the prognosis depends on patient, disease and treatment intertwined factors. The possibility of an immune origin of cytopenia in the advanced stage should be always kept in mind [1].

The diagnosis of AIHA requires the existence of anemia of abrupt onset or significant worsening of previous anemia, increased LDH with or without elevated bilirubin, low or undetectable haptoglobin, positive direct antiglobulin test (DAT) [25,26,27]. The reticulocyte count is usually elevated, although it may be normal in some cases due to poor function of the heavily infiltrated bone marrow or treatment toxicity. The examination of a peripheral blood smear is mandatory because it usually presents spherocytosis and polychromasia [28,29,30].

For the staging of the patient with CLL, a CT scan or an alternative imaging technique should be performed in order to evaluate the possible presence of massive or progressive splenomegaly and/or lymphadenopathies.

Treatment in CLL-associated AIHA is individualized and it depends on the presence of clinical symptoms (acuteness of the onset, grade of anemia and degree of hemolysis) and their severity, disease status and concomitant comorbidities. Anemia, if symptomatic, is an indication for therapy in both newly diagnosed and persistent AIHA [23,31,32].

Typically elderly patients have a lower tolerance to anemia, the treatment of which is therefore frequently required. Furthermore, adverse drug reactions, as well as drug interactions and therapeutic toxicity, are more common in these patients.

RBC transfusions are generally indicated in critical cases with low hemoglobin levels (usually Hb < 6 g/dL) and/or symptomatic anemia, particularly if hemodynamically unstable.

Rituximab is a B-cell depleting monoclonal antibody with CD20 specificity that has demonstrated efficacy in many autoimmune diseases [32,33]. The combined therapy of rituximab and steroids, also administered in first-line treatment, shows a better response than steroid monotherapy [34,35,36]. The addition of rituximab reduces the number of repeated steroid cycles and increases the frequency and duration of response. This is particularly useful in elderly patients with comorbidities. More recently, the targeting of Bruton’s tyrosine kinase (BTK) has markedly improved outcomes for CLL [37].

Cytogenomic analyses are fundamental in the clinical management of hematological malignancies. The European recommendations [38] and the IWCLL guidelines [5] strongly suggest the analysis of multiple loci for the diagnosis and prognostic stratification of patients: deletions in 13q14, 11q22.3/ATM, 17p13.1/TP53, trisomy 12, TP53 mutation and IGHV mutational status. Although not mandatory, conventional cytogenetics using appropriate culture conditions identifies chromosomal abnormalities in the vast majority of cases [39]. A minority of cases present translocations spread across all chromosomes and chromosome bands, primarily as part of a complex karyotype [40].

The present study reports a peculiar CLL case with complex karyotype and refinement of all breakpoints at the gene level by genomic array.

## 2. Case Presentation

A 74-year-old man was admitted to the emergency room following the finding of severe macrocytic anaemia, during blood tests performed that same day for a profound asthenia dating back several months. The anamnesis was silent and the patient was not taking any medications.

The blood examination with Automated Hematology Analyzer DASIT XN-Series showed the data reported in Table 1, whereas the analysis of the lymphocytes subpopulations and the immunophenotyping were performed respectively on AQUIOS CL and NAVIOS EX 3L 10C Beckman Coulter as in Table 2.

The microscopic examination of peripheral blood smears, prepared with the May Grünwald-Giemsa stain (obtained with automated hematology slide preparation unit SP-50; DASIT), revealed atypical lymphocytes, small mature lymphocytes with scant cytoplasm and condensed chromatin that can impart a cracked or “soccer ball” appearance, with the 6% of prolymphocytes, and Gumprecht shadows compatible with a CLL picture. The presence of numerous spherocytes and the finding of a marked polychromasia of the red blood cells correlated well with severe hemolytic anemia and with the consequent conspicuous increase in circulating reticulocytes.

All these findings have advanced the hypothesis of AIHA in lymphoproliferative neoplasia, particularly CLL.

In the patient’s staging a CT scan, an immunophenotyping on peripheral blood and a bone marrow aspiration and biopsy were carried out.

The neck-thorax-abdomen CT scan, performed with and without contrast medium, showed multiple enlarged lymph nodes with oval morphology in all the lymph node stations of the neck. No mediastinal lymphadenomegaly lymphadenomegaly was detected. Besides, bilateral axillary, retroperitoneal and inguinal pathological lymph node enlargements, as well as a homogeneous splenomegaly, were found.

The immunophenotyping showed the presence of a population of CD5+, CD19+, CD20+dim, and CD23+ B lymphocytes with clonal restriction for immunoglobulin lambda surface light-chains, which is proof of monoclonality and indicates malignancy. The immunophenotype was then consistent with CLL-type mature B-cell lymphoproliferative neoplasia (Table 2).

The bone marrow aspiration and the biopsy were performed on the right posterior superior iliac spine. The cellularity consisted, for about 20%, of an infiltrate of small lymphoid elements (Table 2) arranged in perilamellar and centrolacunar aggregates with an interstitial pattern of infiltration. Presence of plasma cells, isolated or in perisinusoidal microaggregates, without evidence of monocatenary restriction and equal to about 5% of bone marrow cellularity, was observed, as well as normo-represented, normo-maturating granulopoietic series. Erythropoietic series was overrepresented compared to granulopoietic series (myeloid series/erythroid series ratio about 1:2), with aspects of topographic dyserythropoiesis. Findings suggest medullary localization of chronic lymphatic leukemia.”

Given the severity of the hemolytic anemia and the incomplete response to corticosteroid therapy, according to ESMO 2021 guidelines, the patient was treated with Rituximab 375 mg/m^2^ weekly for 4 weeks, starting on the fifteenth day from the beginning of the corticosteroid therapy, after reduction of the lymphocyte count below 30,000 lymphocytes/mmc.

At discharge on the sixthteenth day from the start of the corticosteroid therapy, it was performed a biochemical reevaluation that showed a not complete recovery (Table 1).

The patient then continued with the urate-lowering and corticosteroid therapy at home. Because of an asymptomatic COVID-19 infection, the patient had to postpone the subsequent planned dose of Rituximab, but then he could complete the therapeutic scheme (Table 1).

After three months, the patient underwent revaluation. The clinical picture ameliorated with the resolution of AHIA. Due to the complete response to Rituximab and corticosteroid combined therapy and in consideration of the failure of corticosteroid therapy alone, secondary line therapy with Acalabrutinib (Calquence^TM^ 100 mg, 2 tabs/die) was offered to the patient (Table 1).

**Figure 1:**
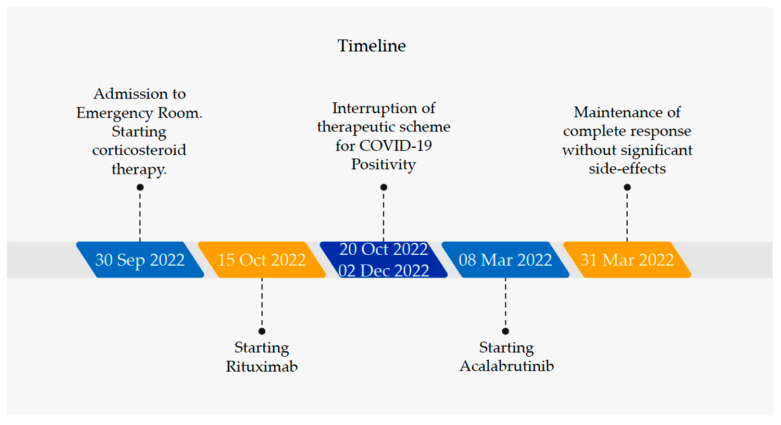
Therapeutic evolution of the patient from diagnosis to complete response based on biochemical and clinical reevaluations.

To these days (i.e., 8 months post patient first admission in the emergency room), the patient has carried out three treatment cycles of secondary line therapy, without significant side effects and with the maintenance of the complete response as regards the hemolytic process (Figure 1).

## 3. Genetic Investigations

**Figure 2:**
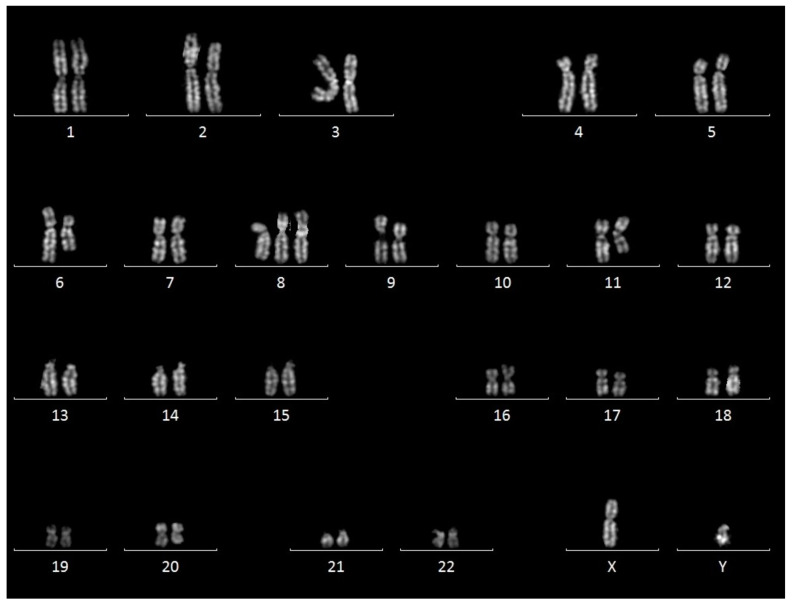
Cytogenetics results. Representative karyogram showing trisomy 8 and aberrant chromosomes 6 and 11.

Conventional karyotyping was performed on peripheral blood cell cultures after 72 h of incubation with appropriate factors for the in-vitro stimulation of B Lymphocytes (ChromoLympho-B proliferation mix, EuroClone). Karyotyping was performed on Quinacrine-stained metaphases using MetaSystems Ikaros software version 5.7. As shown in Figure 2, a complex karyotype was obtained, defined by the presence of >3 numerical and/or structural abnormalities. In fact, the karyotype was characterized by trisomy 8 in 19/20 metaphases and, in all metaphases, an unbalanced translocation between the short arm of chromosome 6 and the long arm of chromosome 11 was detected, concurrent with interstitial deletions in chromosome 6q and 11q that could not be defined in detail.

At first patient re-evaluation, conventional karyotyping from bone marrow cell culture was unchanged as it showed the previously reported unbalanced translocation in all metaphases and trisomy 8 in 5/20 metaphases.

**Figure 3:**
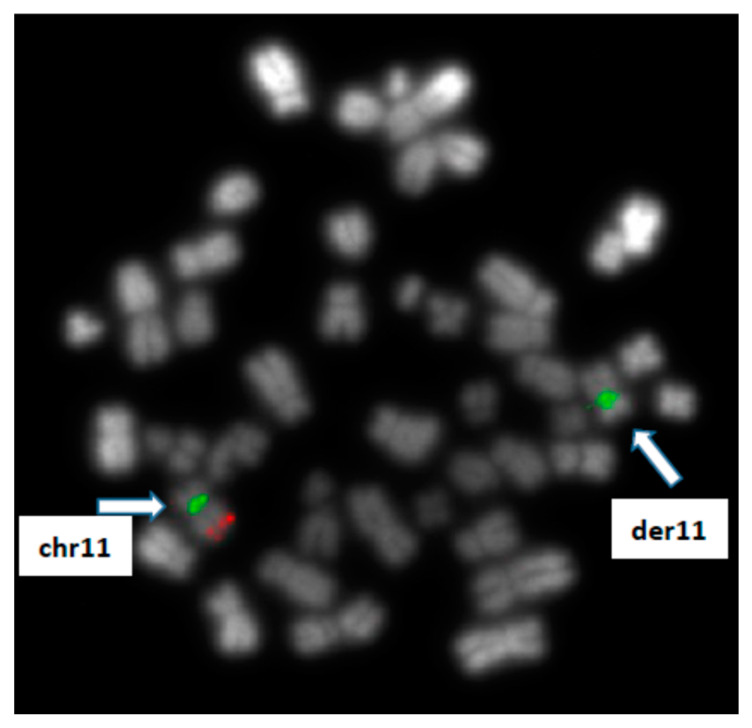
Cytogenetics results: FISH analysis using probes for *ATM* gene (red signal) and chromosome 11 centromere (green signal) revealing a monoallelic loss of ATM on derivative chromosome 11.

In parallel, molecular cytogenetics (FISH) analyses revealed ATM monoallelic deletion (with loss of ATM on derivative chromosome 11) (Figure 3) and retained signals for TP53, 13q14 and centromere 12 FISH probes. The following FISH probes from MetaSystems were used: ATM/cep11, TP53/cep17, RB1/DLEU/LAMP, cep12.

Molecular analysis was performed using Sanger sequencing and did not identify mutations in the *TP53* gene (exons 2–11) and in the variable region of IGH gene *IgHV*.

The aCGH analysis was performed with sex-matched Agilent Reference DNA using the SurePrint G3 CGH + SNP 4 × 180 K slides and the SureScan Microarray Scanner. Number of clones analyzed: 110,712 (CGH) + 59,647 (SNP). Median resolution: 25 kb. Data were analyzed with software CytoGenomics 4.0.3.12; the aberration detection method ADM-2 was applied; the Derivative LogRatio Spread (DLRSD) for this experiment was 0.17 (i.e., optimal quality). Genome positions referred to Human Genome Build 37 (hg19, Assembly February 2009). Aberrations below 50 kb and/or affecting less than five consecutive clones were not reported.

**Figure 4:**
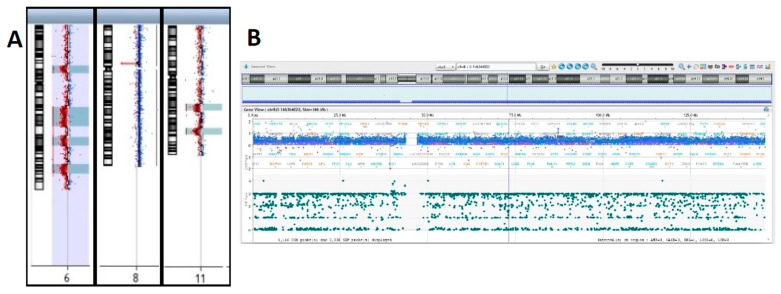

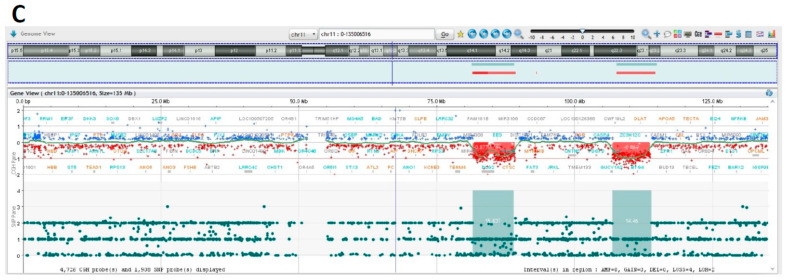
Array-CGH results I. (**A**) Depiction of aberrant chromosomes 6, 8 and 11. Detailed CGH and SNP profiles showing. (**B**) Whole chromosome 8 gain. (**C**) Chromosome 11q deletions.

Array-CGH confirmed trisomy of the entire chromosome 8 (Figure 4A,B) and allowed to resolve in detail of the nature of the unbalanced translocation, revealing multiple regions of genomic losses on chromosomes 6 and 11.

Indeed, one of the deleted regions on chromosome 11 encompasses the ATM locus that showed monoallelic deletion by FISH. The two regions of deletion on chromosome 11 are located in 11q14.1q14.2 and 11q22.3q23.2 (Figure 4C).

**Figure 5:**
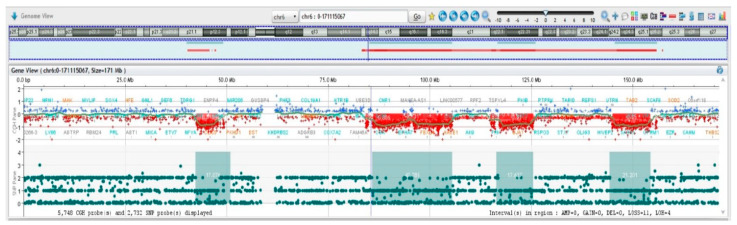
Array-CGH results II. Chromosome 6 has multiple regions of imbalances, indicative of chromotripsis.

The exact boundaries of deletions and the corresponding gene content are listed in Table 2 and Table 3. Interestingly, chromosome 6q showed multiple regions of loss, with eighteen cumulative breakpoints, thus suggesting chromoanagenesis [40,41,42] event as the driving force originating the derivative chromosome 6 (Figure 5, Table 3 and Table S1).

## 4. Discussion

From a genetic point of view, the case under study presented several peculiarities. While common in myeloid disorders, trisomy 8 is very rarely encountered in CLL at diagnosis, usually a mosaic state [43]. Although at mosaic state also in our patient, trisomy 8 represented a prominent cell clone. No signs of myelodysplasia were observed in the peripheral blood or bone marrow aspirate, so that we consider trisomy 8 a secondary, but very early, and positively selected event related to CLL in our patient.

We identified chromosome 11 deletions in bands 11q14.1q14.3, 11q14.3q21 and 11q22.3q23.2 (encompassing the *ATM* gene). As seen in our case, approximately 10–20% of CLL patient exhibit del(11)(q22q23) before treatment [44,45,46,47,48], associated with loss of Ataxia Telangiectasia Mutated (ATM) serine/threonine kinase gene and representing a known marker of worse prognosis. Array-CGH showed multiple regions of deletion on chromosome 6 in our CLL case.

Chromosome 6q deletions are relatively frequent in CLL and are present in approximately 6% of the cases. In recent years, array-CGH analyses allowed to define multiple regions of deletion in 6q and no minimal critical region could be defined so far, as analyses of different case series identified distinct regions of aberration spanning from 6q16 to 6q27 [44,45,46,47,48]. The association between 6q deletion and progression-free survival is conflicting in literature, probably due to the different methodologies applied (FISH, array-CGH, MLPA) and the different 6q bands investigated for prognosis [49,50,51,52]. As a result, the cytogenetic class of poor, intermediate, and good prognosis is associated with 6q deletions in literature. We anticipate that the real impact of distinct 6q deletions in patients’ prognosis and survival will benefit from a widespread application of genomic technologies for patients’ diagnosis. From this point of view, we provide a detailed description of 6q aberrations in our patient including breakpoints and gene content. The array-CGH profile of chromosome 6 is suggestive of a chromoanagenesis event.

In CLL, two-thirds of the cases show no aberrations beyond those detected by the routine FISH panel (TP53, ATM, centromere 12, 13q14) [44]. In fact, CLL shows a low burden of copy-number aberrations, and genomic instability is not considered a prominent feature of CLL [45,53].

In particular, chromoanagenesis is a rare event in CLL, reported in approximately 1–5% of cases analyzed by whole genome sequencing (WGS) or array-CGH [54]. Although we recognize that WGS is the only technique capable to define chromoanagenesis, such event can be inferred also by peculiar genome array profiles. Despite rarely being investigated in clinical trials, there is suggestive evidence that chromoanagenesis may confer worse survival in CLL [55]. In our case, the elevated number (eighteen) of breakpoints in the long arm of chromosome 6 fulfills the criteria for a bona fide chromotripsis-like event. Notably, in our patient, this event is not associated with TP53 alterations.

## 5. Conclusions

We report the genetic findings of a CLL patient with abrupt disease onset, so far responding properly to treatments despite the presence of distinct genetic adverse traits including ATM deletion, complex karyotype and chromosome 6q chromoanagenesis event. Our report confirms that interphase FISH alone is not able to provide an overview of the whole genomic landscape in selected CLL cases and that additional techniques are required to reach an appropriate cytogenetic stratification of patients.

## Figures and Tables

**Table 1:** Blood examination, biochemical analysis and drugs administration evolution.

	30 September 2022	15 October 2022	24 October 2022	9 December 2022	16–23 December 2022	8 March 2022	Normal Range	SI
WBC	143,960	4130	4020	6770			4000–9500	10^9^/L
Hb	52	69	91	136			130–170	g/L
MCV	119.6	128.8	125.4	93.0			80.0–95.0	fL
PLT	149,000	120,000	104,000	215,000			130,000–400,000	10^9^/L
ANC	4600	1970	2400	4080				10^9^/L
ALC	129,320	2010	1410	2220				10^9^/L
AMC	9480	110	200	430				10^9^/L
HP	<20.00						50–150	mG/dL
sCr	1.10	0.86		0.94			<1.25	mG/dL
TBILI	3.70			0.50			0.20–1.30	mG/dL
IBILI	3.40							mG/dL
LDH	337			355			120–246	IU/mL
CRP	5.00			5.00			<10	mG/L
Ret	142.2							10^9^/L
Treatment Administration	Corticosteroid	Rituximab	Rituximab interruption for COVID Positivity (20 October 2022–2 December 2022)	Rituximab	Rituximab	Acalabrutinib		

Abbreviations: International System of Units (SI); White Blood Cell count (WBC); Haemoglobin (Hb); Mean Cell Volume (MCV); Platelet count (PLT); Absolute Neutrophil Count (ANC); Absolute Lymphocyte Count (ALC); Absolute Monocyte Count (AMC); Haptoglobin (HP); sieric Creatinine (sCr); Total Bilirubin (TBILI); Indirect Bilirubin (IBILI); Lactate Dehydrogenase (LDH); C-Reactive Protein (CRP) mg/L; Reticulocite (Ret); Liter (L); Grams (g); Femtoliter (fL); Milligram (mG); Deciliter (dL); International Unity (IU).

**Table 2:** Comparison between the immunophenotyping on peripheral blood and bone marrow.

	Peripheral Blood	Bone Marrow
	CD5+	CD5+
	CD20+dim	CD20+
	CD23+	CD23+
	CD19+	CD3-
	LAMBDA+	LEF1+
		CYCLIN D1-
Conclusion	CLL-type mature B-cell lymphoproliferative neoplasia	Medullary localization of CLL

**Table 3:** Genetic investigations and results.

Karyotyping	47,XY,+8,der(6)t(6;11)(p12;q24)del(6)(q14q22),der(11)t(6;11)(p12;q24)del(11)(q13q24)[19]/ 46,XY,der(6)t(6;11)(p12;q24)del(6)(q14q22),der(11)t(6;11)(p12;q24)del(11)(q13q24)[1]	
FISH	Monoallelic deletion of ATM in 90% of nuclei	
	Retained signal of TP53, 13q14, centromere 12	
Sequencing	TP53 and IGHV wild type	
Array-CGH result arr[GRCh37]	Copy number variation	Size (Mb)
	6p21.1p12.3(42530647_47909775) × 1	5.4 Mb
	6p12.3(48415298_48509357) × 1	0.1 Mb
	6p12.3(49041219_49391971) × 1	0.35 Mb
	6q14.2(84136451_84871569) × 1	0.73 Mb
	6q14.3q21(85872402_105528935) × 1	19.6 Mb
	6q22.1(114960420_116038498) × 1	1 Mb
	6q22.1q22.31(117082970_125486670) × 1	8.4 Mb
	6q24.2q25.2(144831439_154339818) × 1	9.5 Mb
	6q25.3(155552589_155880666) × 1	0.33 Mb
	arr[GRCh37] (8) × 3	arr[GRCh37] (8) × 3
	11q14.1q14.3(81540019_89188979) × 1	7.65 Mb
	11q14.3q21(92716797_92927742) × 1	0.21 Mb
	11q22.3q23.2(106853105_113669303) × 1	6.8 Mb

Even in the first genetic revaluation, we obtained the same results.

## Data Availability

We state the data are available to the scientific community.

## Supplementary Materials

The following supporting information can be downloaded at: https://www.mdpi.com/article/10.3390/jcm12124110/s1, Table S1: Gene content of the genomic regions of deletion identified by array-CGH.
