# Supplementary material for: A Peculiar CLL Case with Complex Chromosome 6 Rearrangements and Refinement of All Breakpoints at the Gene Level by Genomic Array: A Case Report
Source: J Clin Med. 2023 Jun 17;12(12):4110. doi: 10.3390/jcm12124110 (PMC10299087; doi:10.3390/jcm12124110)
Supplement: Supplementary file 1 [file jcm-12-04110-s001.zip › jcm-2401007-supplementary.pdf]

Table S1. Gene content of the genomic regions of deletion identified by array-CGH.

ISCN Nomenclature arr[GRCh37]	Mb	Genes Deleted
6p21.1p12.3(42530647_47909775)x1	5.4	UBR2, PRPH2, TBCC, PTCRA, CNPY3, GNMT, PEX6, PPP2R5D, MEA1, KLHDC3, RRP36, CUL7, MRPL2, PTK7, SRF, CUL9, SLC22A7, ABCC10, TJAP1, YIPF3, POLR1C, XPO5, POLH, GTPBP2, RSPH9, MRPS18A, MRPL1, CAPN11, SLC29A1, HSP90AB1, SLC35B2, NFKBIE, TCTE1, AARS2, CDC5L, SUPT3H, RUNX2, CLIC5, RCAN2, CYP39A1, SLC25A27, TDRD6, PLA2G7, MEP1A, CD2AP, OPN5
6p12.3(48415298_48509357)x1	0.1	---
6p12.3(49041219_49391971)x1	0.35	---
6q14.2(84136451_84871569)x1	0.73	ME1, SNAP91, RIPPLY2, CYB5R4, MRAP2, CEP162, PRSS35
6q14.3q21(85872402_105528935)x1	19.6	NT5E, SNX14, SYNCRIP, SNHG5, SNORD50A, SNORD50B, HTR1E, CGA, ZNF292, GJB7, SLC35A1, RARS2, ORC3, AKIRIN2, SPACA1, CNR1, RNGTT, PNRC1, PM20D2, GABRR1, GABRR2, UBE2J1, RRAGD, ANKRD6, CASP8AP2, GJA10, BACH2, MAP3K7, EPHA7, MANEA, FUT9, UFL1, FHL5, GPR63, NDUFAF4, MMS22L, POU3F2, FBXL4, PNISR, CCNC, PRDM13, MCHR2, SIM1, ASCC3, GRIK2, HACE1, LIN28B, USP45, COQ3
6q22.1(114960420_116038498)x1	1	---
6q22.1q22.31(117082970_125486670)x1	8.4	RFX6, VGLL2, ROS1, GOPC, NUS1, PLN, MCM9, ASF1A, MAN1A1, TBC1D32, GJA1, HSF2, SERINC1, PKIB, FABP7, SMPDL3A, TRDN, NKAIN2, TPD52L1, GPRC6A, CEP85L, CLVS2, RNF217
6q24.2q25.2(144831439_154339818)x1	9.5	UTRN, EPM2A, FBXO30, GRM1, RAB32, ADGB, STXBP5, SASH1, UST, TAB2, SUMO4, ZC3H12D, PPIL4, KATNA1, LATS1, NUP43, PCMT1, ULBP2, ULBP1, RAET1L, ULBP3, PPP1R14C, IYD, MTHFD1L, ZBTB2, RMND1, ARMT1, ESR1, SYNE1, MYCT1, VIP, FBXO5, OPRM1, SHPRH, RAET1E, RAET1G, RAET1K, PLEKHG1, AKAP12, MTRF1L, RGS17
6q25.3(155552589_155880666)x1	0.33	TIAM2, TFB1M, NOX3
11q14.1q14.3(81540019_89188979)x1	7.65	PRCP, RAB30, PCF11, DLG2, TMEM126B, TMEM126A, SYTL2, PICALM, EED, C11orf73, ME3, FZD4, TMEM135, RAB38, CTSC, GRM5, TYR, NOX4,DDIAS, ANKRD42, PRSS23
11q14.3q21(92716797_92927742)x1	0.21	MTNR1B, SLC36A4
11q22.3q23.2(106853105_113669303)x1	6.8	GUCY1A2, ALKBH8, ELMOD1, SLN, CUL5, ACAT1, NPAT, ATM, EXPH5, DDX10, ZC3H12C, RDX, FDX1, COLCA1, COLCA2, POU2AF1, BTG4, MIR34B, SIK2, PPP2R1B, ALG9, CRYAB, HSPB2, DIXDC1, DLAT, SDHD, IL18, TEX12, PTS, NCAM1, TTC12, ANKK1, DRD2, TMPRSS5, ZW10, USP28, RAB39A, ARHGAP20, LAYN, TIMM8B, BCO2, PLET1, CLDN25
